# S1P1 Threonine 236 Phosphorylation Mediates the Invasiveness of Triple-Negative Breast Cancer and Sensitivity to FTY720

**DOI:** 10.3390/cells12070980

**Published:** 2023-03-23

**Authors:** Fabrice J. F. Laroche, Sheng Li, Ning Shen, Soo Kyung Hwang, Gina Nguyen, Wenling Yu, Chen Khuan Wong, Ryan J. Quinton, Jason N. Berman, Ching-Ti Liu, Anurag Singh, Neil J. Ganem, Sam Thiagalingam, Hui Feng

**Affiliations:** 1 Departments of Pharmacology and Medicine, Section of Hematology and Medical Oncology, Chobanian & Avedisian School of Medicine, Boston University, Boston, MA 02118, USA; 2 Institute of Agro-Bioengineering and College of Life Sciences, Guizhou University, Guizhou 550025, China; 3 Biomedical Genetics Section, Department of Medicine, Department of Pathology and Laboratory Medicine, Genetics and Genomics Graduate Program, Cancer Center, Chobanian & Avedisian School of Medicine, Boston University, Boston, MA 02118, USA; 4 Children’s Hospital of Eastern Ontario Research Institute, Departments of Pediatrics and Cellular and Molecular Medicine, University of Ottawa, Ottawa, ON K1H 8L1, Canada; 5 Department of Biostatistics, School of Public Health, Boston University, Boston, MA 02118, USA

**Keywords:** triple-negative breast cancer, sphingosine 1-phosphate receptor 1, threonine 236 phosphorylation, invasiveness, zebrafish, MK2206, FTY720

## Abstract

Hyperactive sphingosine 1-phosphate (S1P) signaling is associated with a poor prognosis of triple-negative breast cancer (TNBC). Despite recent evidence that links the S1P receptor 1 (S1P1) to TNBC cell survival, its role in TNBC invasion and the underlying mechanisms remain elusive. Combining analyses of human TNBC cells with zebrafish xenografts, we found that phosphorylation of S1P receptor 1 (S1P1) at threonine 236 (T236) is critical for TNBC dissemination. Compared to luminal breast cancer cells, TNBC cells exhibit a significant increase of phospho-S1P1 T236 but not the total S1P1 levels. Misexpression of phosphorylation-defective *S1P1 T236A* (alanine) decreases TNBC cell migration in vitro and disease invasion in zebrafish xenografts. Pharmacologic disruption of S1P1 T236 phosphorylation, using either a pan-AKT inhibitor (MK2206) or an S1P1 functional antagonist (FTY720, an FDA-approved drug for treating multiple sclerosis), suppresses TNBC cell migration in vitro and tumor invasion in vivo. Finally, we show that human TNBC cells with AKT activation and elevated phospho-S1P1 T236 are sensitive to FTY720-induced cytotoxic effects. These findings indicate that the AKT-enhanced phosphorylation of S1P1 T236 mediates much of the TNBC invasiveness, providing a potential biomarker to select TNBC patients for the clinical application of FTY720.

## 1. Introduction

Breast cancer is the second leading cause of mortality in women worldwide [1]. This heterogeneous disease consists of four subtypes ranked in order of increasing aggressiveness: luminal A, luminal B, HER2, and basal-like/triple-negative [2]. The effectiveness of current treatments for breast cancer depends largely on the expression of hormone receptors for estrogen (ER), progesterone (PR), or the overexpression/amplification of human epidermal growth factor receptor-2 (HER2). Triple-negative breast cancer (TNBC) is often diagnosed in young women, spreads rapidly, and confers an inferior survival rate with the majority of deaths occurring within the first five years of detection [3]. Lacking expression of the ER, PR, or HER2 receptors, TNBC is treated with an array of cytotoxic agents, either administered alone or with surgery and/or radiotherapy. Although patients respond well initially and have recently been offered immunotherapy [4,5], the prognosis for TNBC remains poor due to the course of the aggressive disease and the lack of predictive biomarkers to guide therapeutic selection [6,7].

Hyperactive sphingosine 1-phosphate (S1P) signaling can induce cancer progression in both solid and hematological malignancies [8]. Produced through the phosphorylation of sphingosine by sphingosine kinases SPHK1 or SPHK2, S1P functions either intracellularly as a secondary messenger or is exported from cells to act as a ligand for a family of five G protein-coupled receptors (GPCR; namely, S1P1, S1P2, S1P3, S1P4, and S1P5). Depending on the cell type, the S1P receptor, and the G protein subtype, extracellular S1P signaling enhances a wide range of cellular activities, including motility, survival, and angiogenesis. Aberrant activation of this signaling pathway is often caused by an increased expression of the SPHK1 and/or S1P receptors [9]. Indeed, SPHK1 was found to be upregulated in TNBC and associated with a poor overall and progression-free survival rate [10]. FTY720, an FDA-approved drug for treating multiple sclerosis, is a functional antagonist for S1P receptors and exhibits anti-TNBC activity when combined with EGFR inhibitors in a murine model [11]. As a single agent, FTY720 exhibits variable antitumor efficacy, ranging from no to good responses depending on the TNBC cells tested. However, it remains unknown what molecular properties of TNBC cells will confer on them to FTY720 sensitivity. 

Among the five S1P receptors, S1P1 has gained attention as the main receptor responsible for vascular development and immune cell regulation [12]. Originally known as endothelial differentiation gene 1 (*EDG1*), *S1P1* was discovered as one of the immediate early-regulatory genes of endothelial cell differentiation [13]. As a GPCR, it couples with the Gi protein and promotes cell survival, angiogenesis, and motility through one or more of its downstream effectors, such as ERK-1/2, PI3K/AKT, JNK, and RAC [9]. Interestingly, AKT can also serve as an upstream regulator to phosphorylate S1P1 at the threonine 236 residue (T236), leading to endothelial cell migration [14]. S1P1 has also been shown to regulate lymphocyte migration and promote normal embryonic nervous system development [15,16]. In addition to its normal functions, S1P1 contributes to multiple human cancers, stimulating tumor cell migration and invasion in Wilms tumor, fibrosarcoma, and glioblastoma [17,18,19]. Furthermore, S1P1 is upregulated in the blood vessels of Lewis lung carcinoma, while its knockdown inhibits vascular stabilization, angiogenesis, and tumor growth [20]. In the context of breast cancer, high S1P1 levels correlate with rapid recurrence rates, shorter survival times, and increased resistance to tamoxifen in ER-positive breast cancer patients [21]. Emerging evidence revealed that several experimental treatments can kill TNBC by downregulating S1P1 signaling, suggesting its importance in TNBC survival [22,23]. Hence, it is crucial to apply animal models to gain an in-depth understanding of this receptor in TNBC pathogenesis to guide therapeutic selection.

Here we combine the genetic and biochemical analyses of human TNBC cells with real-time monitoring of tumor cell dissemination in zebrafish xenografts to understand the role and mechanisms of action of S1P1 in TNBC invasiveness. Unlike luminal breast cancer, S1P1 expression was not elevated in human TNBC cells. Instead, we found that AKT-mediated phosphorylation of S1P1 at T236 was the factor most closely related to TNBC invasiveness and distant spread. In addition, FTY720 was sufficient in blocking tumor cell dissemination while inducing cell death for TNBC with an elevated expression of phospho-S1P1 T236 (P-S1P1 T236).

## 2. Materials and Methods

### 2.1. Zebrafish Maintenance and Husbandry

Zebrafish husbandry was performed as described in the aquatic facility at the Boston University School of Medicine, in accordance with our IACUC-approved protocol. Zebrafish lines used in this study include *AB*, *Casper*, and *Casper;Tg*(*fli1:EGFP*).

### 2.2. Subcloning

The human *S1P1* cDNA was mutated at the threonine (T) 236 site to alanine (A) using polymerase chain reaction (PCR) for site-directed mutagenesis with the QuickChange II Kit (Agilent). Both *S1P1* wild-type and *T236A* mutant sequences were amplified by PCR and cloned into the *PDONR221* gateway entry vector (Thermo Fisher Scientific, Waltham, MA, USA). These entry plasmids were subsequently recombined with the *PWPI-DEST-GFP* bicistronic lentiviral vector, thus creating PWPI-DESTGFP-S1P1-WT and *PWPI-DEST-GFP-S1P1-T236A* overexpression plasmids. The *PLemiR-RFP* lentiviral vector was used for fluorescence tracking of cell lines upon viral infection.

### 2.3. Cell Line Maintenance and Lentivirus Transduction

All cell lines used in this study were cultivated according to ATCC recommendations. For virus production, the human embryonic kidney cells (HEK293T) were incubated with the gene-of-interest plasmid, psPAX2, and pMD2.G lentiviral vectors using serum-free OPTI-MEM (Thermo Fisher Scientific) according to standard procedure in the presence of polybrene (Millipore, Burlington, MA, USA). HEK293T supernatant containing each type of lentivirus (*RFP*, *Luciferase*, *S1P1 WT*, or *S1P1 T236A*) was used to infect MCF10A-derived cell lines. Cells were subsequently sorted based on GFP or RFP expression. Overexpression of *S1P1* was verified using western blotting.

### 2.4. Cell Proliferation and Wound-Scratch Assays

Equal numbers of cells were seeded into 96-well plates (500 cells per well for proliferation assays and 5000–10,000 cells per well for viability assays) or 6-well plates (500,000 cells per well for wound healing assays), respectively, in triplicates. For the proliferation and viability assays, Cell Titer blue kits (Promega, Madison, WI, USA) were used according to manufacturer instructions in order to quantify live cells. For wound healing assays, scratches were performed using a pipet tip, washed once with PBS, and replaced with a full growth medium. Images were acquired using an EVOS Xl microscope with a 4× objective (Thermo Fisher Scientific), either directly after the scratch or over time to monitor wound recovery.

### 2.5. Zebrafish Transplantation and Imaging

The 48-hpf embryos were dechorionated enzymatically using Pronase (Roche Diagnostics Corporation, Indianapolis, IN, USA) and anesthetized with tricaine (Western Chemical Inc., Ferndale, MA, USA). Human TNBC cells were trypsinized, washed, counted, and resuspended to obtain a final concentration of 10 × 10^6^ cells per mL in a complete growth media. All cells were either expressing RFP or stained with Cell tracker Orange CMTMR (Thermo Fisher Scientific). About 1 nL of these cells was microinjected into the vascularized area near the perivitelline cavity of the embryo using a needle holder and a microinjection station (World Precision Instruments, Sarasota, FL, USA). These zebrafish xenografts were incubated in the dark at 35 °C for three days and monitored by fluorescence imaging to document tumor cell survival and metastatic spreads. Zebrafish embryos were immobilized using low-temperature melting agarose (Fisher Bioreagents, Pittsburgh, PA, USA) for acquiring images or movies using an MVX10 stereomicroscope (Olympus of America). The metastatic cells were quantified using *Fiji* software based on fluorescent intensity outside the injection area (ImageJ2) or, in some experiments, counted manually.

### 2.6. Small Molecule Treatment

For viability assays, 5000 to 10,000 cells per well were seeded in a 96-well plate with a media containing MK2206 (Cayman Chemical, Ann Arbor, MI, USA) or FTY720 (Cayman Chemical) for two days, using cells treated with DMSO (Cayman Chemical) alone as controls. Cell viability was measured by Cell Titer blue assay (Promega) and was presented as a percentage of control cells. Zebrafish transplanted with human TNBC cells were incubated in the presence of MK2206 at 0.3 μM or FTY720 at 2 μM in fish water for three days, using embryos treated with DMSO as controls. Fish water containing the compound was refreshed daily.

### 2.7. Protein Extraction and Western Blotting

Breast cancer cells were collected and lysed in RIPA buffer supplemented with Halt proteinase and phosphatase inhibitor cocktail (Thermo Fisher Scientific). The primary antibodies included anti-SPHK1 (Cell signaling Technology, Danvers, MA, USA), anti-EDG1/S1P1 (Santa Cruz Biotechnology, Dallas, TX, USA), antiphospho-EDG1/S1P1 T236 (Abcam, Cambridge, UK), anti-AKT and anti-phospho-AKT S473 (Cell signaling Technology), anti-active-Caspase 3 (Cell signaling Technology), and anti-β-ACTIN (Sigma-Aldrich, St. Louis, MO, USA). Secondary antibodies included horseradish peroxidase-conjugated anti-mouse or anti-rabbit antibodies (Pierce, Waltham, WA, USA). Chemiluminescent images were obtained using a *G:BOX* Chemi XT4 (Syngene, Bangalore, India) and quantified with Fiji software.

### 2.8. Gene Expression Analysis

The dataset (GSE2034) for human breast cancer patient samples was obtained from Gene Expression Omnibus (GEO) [24]), and reanalyzed using R studio (3.3.2) for differential gene expression. We classified the 426 patients in GSE2034 dataset into two subtypes: TNBC and non-TNBC. We used *ER* (*ESR1*) ≥ 1000, *PR* (*PGR*) ≥ 20, *HER2* (*ERBB2*) ≥ 3700 as thresholds, and the data less than the three thresholds were considered as TNBC (*n* = 41), while the rest were classified as non-TNBC (*n* = 385).

### 2.9. Statistical Analysis

GraphPad Prism software was used to calculate significant values (*p*) using a two-tailed Student’s *t*-test for the probes of interest in gene expression analysis, protein-of-interest to ACTIN ratio for western blot analysis, tumor burden, migration, and invasive spread of TNBC cells. The false discovery rate (FDR) was used to test all the probes in gene expression analysis. The *p*-values or the FDR-adjusted *p*-value (q-value) less than or equal to 0.05 were considered statistically significant.

## 3. Results

### 3.1. T236 Phosphorylation but Not Total S1P1 Is Elevated in Human TNBC Cells

**Figure 1:**
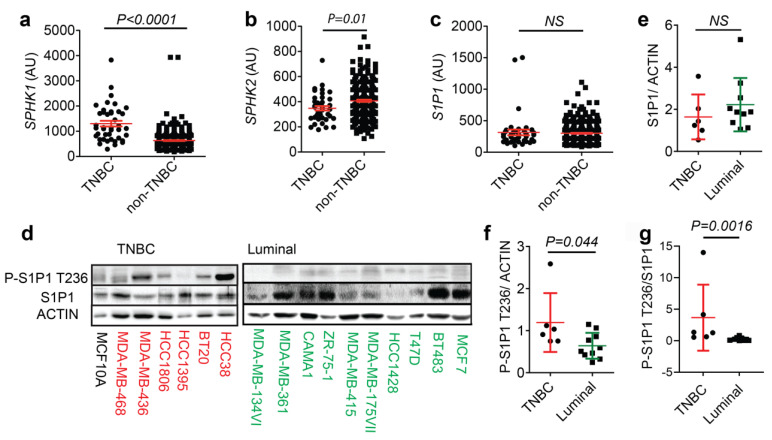
Phospho-S1P1 T236 Levels, but not Total S1P1, are Elevated in Human TNBC Cells. (**a**–**c**) Relative transcript levels of *SPHK1* (**a**), *SPHK2* (**b**), and *S1P1* (**c**) in human TNBC (*n* = 41) and non-TNBC samples (*n* = 385) from a reanalyzed microarray dataset (GSE2034; [24]). (**d**) Western blot analysis of phospho-S1P1 T236 (P-S1P1 T236), total S1P1, and ACTIN levels in human TNBC (*n* = 6) and luminal (*n* = 10) cell lines with MCF10A nontransformed cell line as a control. (**e**–**g**) S1P1 (**e**) and P-S1P1 T236 (**f**) to ACTIN as well as P-S1P1 T236 to S1P1 ratio (**g**) in TNBC and luminal breast cancer cell lines. Data indicate mean ± SEM.

To understand how the S1P signaling pathway contributes to TNBC pathogenesis, we first performed a comparative analysis of gene expression in the sphingosine lipid metabolism pathway, using a publicly available database for primary breast cancer patient samples (GSE2034) [24]. We found a significantly higher expression of *SPHK1* mRNA in TNBC compared to non-TNBC patient samples (Figure 1a). By contrast, *SPHK2* was clearly downregulated in the same samples (Figure 1b). Interestingly, there were no significant differences in the mRNA expression of *S1P1* between TNBC and non-TNBC patient samples (Figure 1c).

Given the recent findings that S1P1 is important for TNBC survival [22,23], we next performed a Western blot analysis to assess the protein levels of S1P1 in a panel of human TNBC and less aggressive luminal breast cancer cell lines, as well as a nontransformed breast epithelial cell line (MCF10A) (Figure 1d). Consistent with our transcript findings, we did not detect significant differences in total S1P1 protein levels between TNBC and luminal breast cancer cells (Figure 1e). Rather, P-S1P1 T236 levels were significantly higher in TNBC than in luminal breast cancer cells (Figure 1f,g). Among the TNBC cell lines, the highly metastatic MDA-MB-436 and HCC38 cells showed the most pronounced phosphorylation of S1P1 T236. Together, these results demonstrate increased levels of phospho-S1P1 T236 but not total S1P1 in highly invasive TNBC cells, relative to those in less aggressive luminal breast cancer cells.

### 3.2. TNBC Progression Positively Correlates with S1P1 T236 Phosphorylation

**Figure 2:**
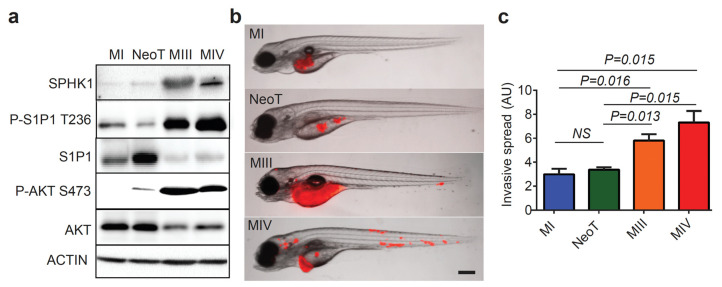
Increased Levels of P-S1P1 T236 are linked with TNBC Progression. (**a**) Western blot analysis of SPHK1, P-S1P1 T236, S1P1, phospho-AKT S473 (P-AKT S473), AKT, and ACTIN levels in human MI, NeoT, MIII, and MIV cells. (**b**) Overlay of brightfield and fluorescence images of zebrafish embryos transplanted with RFP^+^ human MI, NeoT, MIII, and MIV cells at 3 days post-transplantation (dpt; *n* = 3 per group). The scale bar represents 200 µm. (**c**) Quantification of invasive spread in zebrafish embryos based on fluorescence intensity of MI, NeoT, MIII, and MIV cells (*n* = 3 per group). Data presented as Mean ± SEM. NS, not significant.

Having identified a link between the phosphorylation of S1P1 T236 and human TNBC cells, we sought to clarify how the phosphorylation of S1P1 T236 might contribute to disease aggressiveness. We utilized the well-characterized MCF10A-derived model of TNBC progression, consisting of four basal-like/TNBC cell lines with increasing degrees of aggressiveness: nontransformed MCF10A (MI), transformed benign MCF10AT (NeoT), malignant and metastatic MCF10CA1h (MIII), and the most aggressive and invasive MCF10CA1a.cl1 (MIV) [25]. Western blot analysis revealed that SPHK1 and phosphorylated S1P1 T236 and AKT serine 473 (S473) were progressively increased in the more aggressive MIII and MIV TNBC cells, compared with nontransformed MI and benign NeoT cells (Figure 2a). In contrast, total S1P1 and AKT expression appeared to be downregulated in MIII and MIV cells.

It has been well demonstrated that zebrafish xenografts can mirror the metastatic properties of human cancer cells in vitro and in mice [26]. Moreover, the transparency and rapid development of the zebrafish enable real-time monitoring of cancer invasion and dissemination in vivo in days rather than weeks to months when using murine models [27,28,29,30]. Hence, we transplanted fluorescently labeled human MCF10A-derived cells into zebrafish embryos at 48 h postfertilization (hpf) to further assess the pathogenic contribution of P-S1P1 T236. At three days post-transplantation (dpt), no distant dissemination of MI and NeoT cells was observed, whereas MIII and MIV cells showed pronounced spread throughout the zebrafish (Figure 2b). Quantification of the individual disseminated cells indicated a significantly more distant spread of MIII and MIV cells compared to MI and NeoT (Figure 2c). Furthermore, real-time fluorescence imaging and time-lapse movies of zebrafish xenografts demonstrated intravasation and active circulation of MIII cells, though not MI or NeoT cells, at three dpt (Figure S1a and Movie S1). Despite similar tumor burdens introduced (Figure S1b), transplanted MIII and MIV cells significantly decreased the viability of the zebrafish embryos compared to those injected with MI and NeoT cells (Figure S1c). These findings suggest that high levels of P-S1P1 T236 are linked with the greater invasive potential of human TNBC cells in vivo.

### 3.3. Phosphorylation of S1P1 T236 Is Essential for TNBC Migration and Invasion

**Figure 3:**
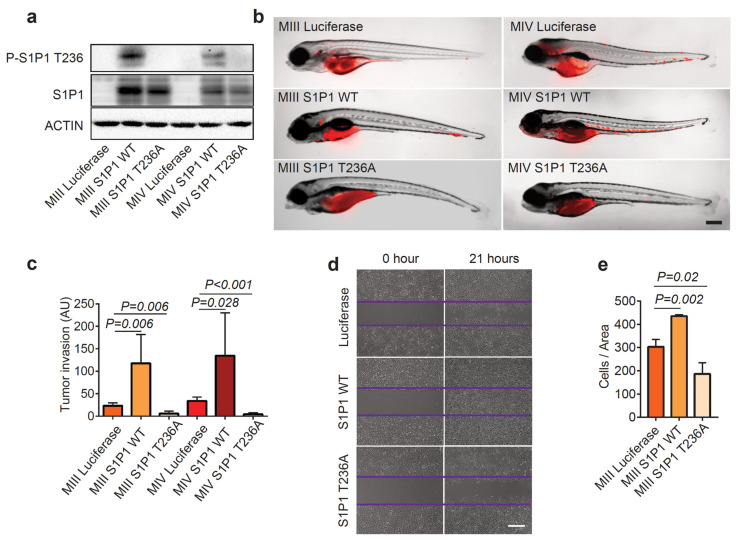
P-S1P1 T236 contributes to the distant spread of TNBC. (**a**) Western blotting analysis of P-S1P1 T236, S1P1, and ACTIN in MIII and MIV cells overexpressing Luciferase, S1P1 WT, or the phosphorylation-defective S1P1 T236A mutant. (**b**) Overlay of brightfield and red fluorescence images of zebrafish xenografts transplanted with RFP^+^ MIII and MIV cells overexpressing *Luciferase*, *S1P1 WT*, or *S1P1 T236A* at 3 dpt (right panel). (**c**) Quantification of invasive properties of MIII and MIV cells overexpressing *Luciferase*, *S1P1 WT*, or *S1P1 T236A* in zebrafish xenografts based on fluorescence intensity of RFP^+^ tumor cells outside of the initial injection area (*n* = 6 for MIII and MIV overexpressing *Luciferase*, and *n* = 3 for MIII and MIV overexpressing *S1P1 WT* or *S1P1 T236A*). (**d**,**e**) Wound-scratch assay (**d**) and quantification (**e**) show increased migration properties of MIII cells overexpressing *S1P1 WT* though not *S1P1 T236A* at 21 h after wound scratch, compared to control Luciferase-overexpressing cells (*n* = 3 per group). Data are presented as Mean ± SEM. Scale bar = 200 µm in (**b**) and Scale bar = 5 μM in (**d**).

To investigate whether phosphorylation of S1P1 T236 directly mediates TNBC aggressiveness, we evaluated the effects of overexpressing *S1P1* wild-type (*WT*) or phosphorylation-defective mutant (*T236A*) on MIII and MIV cell behavior in zebrafish xenografts. A western blot analysis demonstrated elevated S1P1 and P-S1P1 T236 levels in *S1P1*-overexpressing MIII and MIV cells, compared to control *Luciferase*-overexpressing cells (Figure 3a). Despite a similar tumor burden (Figure S2a), we observed greater dissemination of the MIII and MIV cells overexpressing *S1P1 WT* throughout the zebrafish at three dpt, compared to the control cells (Figure 3b,c). Strikingly, overexpression of the *S1P1 T236A* mutant significantly inhibited the distant spread of the human TNBC cells in zebrafish—the opposite effect from that seen with *S1P1 WT* overexpression (Figure 3b,c). Quantification of disseminated cells demonstrated a significant reduction in the distant spread of MIII and MIV cells overexpressing *S1P1 T236A*, compared to cells overexpressing either *S1P1 WT* or *Luciferase* (Figure 3c).

To understand how increased phosphorylation of S1P1 T236 impacts TNBC invasiveness, we measured the proliferation rate of MIII cells overexpressing *Luciferase*, *S1P1 WT*, or *S1P1 T236A*. Compared to the controls, overexpressing neither *S1P1 WT* nor *S1P1 T236A* affected MIII cell proliferation (Figure S2b). Given that AKT-mediated phosphorylation of S1P1 T236 can promote endothelial cell migration [14], we considered that S1P1 T236 phosphorylation might be required for the migration of TNBC cells. Thus, we performed wound-scratch assays with MIII cells overexpressing *Luciferase*, *S1P1 WT*, or *S1P1 T236A* (Figure 3d). Our data revealed that overexpression of *S1P1 WT* in MIII cells led to increased levels of P-S1P1 T236 and more rapid closure of the wound gap than seen in controls (Figure 3a,d,e). By contrast, overexpression of the dominant-negative phosphorylation-defective *S1P1 T236A* mutant in MIII cells significantly inhibited the healing of the wound gap (Figure 3a,d,e). These results indicate that phosphorylation of S1P1 T236 is essential for TNBC invasion and distant dissemination due to their enhanced migration.

### 3.4. AKT Phosphorylates S1P1 T236 in TNBC Cells to Promote Disease Invasion

**Figure 4:**
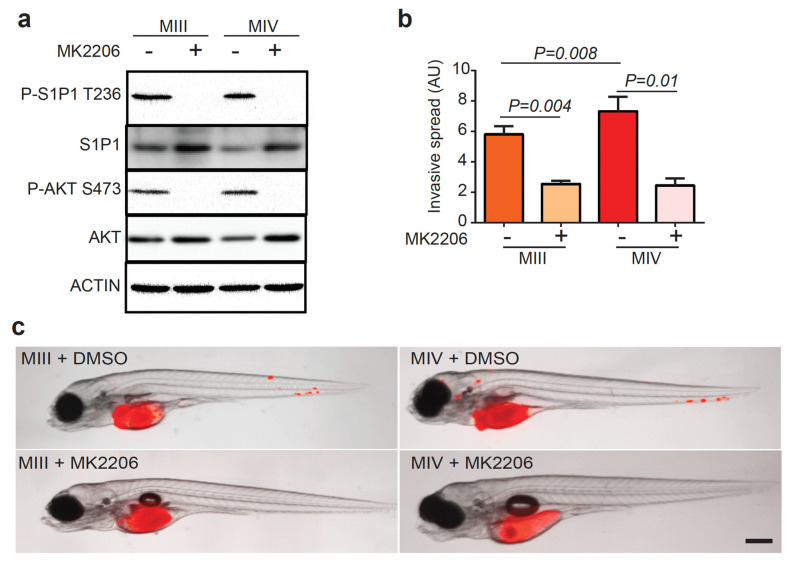
AKT Inhibitor MK2206 reduces P-S1P1 T236 and impairs TNBC invasion in Zebrafish xenografts. (**a**) Western blot analysis of P-S1P1 T236, S1P1, P-AKT S473, AKT, and ACTIN in MIII and MIV cells without and with MK2206 (90 nM) treatment. (**b**) Quantification of fluorescence intensity of tumor burden in (**c**, *n* = 3 per group). (**c**) Overlay of brightfield and fluorescence images of zebrafish embryos transplanted with RFP^+^ MIII and MIV cells and treated with DMSO or MK2206 (0.3 μM) at 3 dpt. Scale bar = 200 µm. Data are presented as Mean ± SEM.

AKT binds directly to the third intracellular loop of the S1P1 receptor and phosphorylates its T236 residue in endothelial cells [14]. To determine if this mechanism also operates in TNBC cells, we treated the MCF10A series with MK2206, a potent allosteric pan-AKT inhibitor tested in clinical trials for treating advanced-stage solid tumors. Western blot analysis of MIII and MIV cells showed that a low dose of MK2206 (90 nM) effectively inhibited AKT S473 phosphorylation (Figure 4a), while total AKT and S1P1 expression appeared slightly upregulated. Surprisingly, the treatment of the four MCF10A series of cell lines with MK2206, ranging from low to high concentrations (up to 30 μM), did not reduce cell viability after 48 h of treatment (Figure S3), a result consistent with an early report on MCF10A by others [31]. To determine if AKT is responsible for the S1P1-mediated invasion of human TNBC cells in vivo, we monitored the dissemination of MIII and MIV cells treated with MK2206 in zebrafish embryos (Figure 4b,c). Despite similar tumor burdens introduced (Figure S4a), MK2206 significantly suppressed the distant spread of MIII and MIV cells at three dpt, compared to vehicle-treated control cells (Figure 4b,c).

**Figure 5:**
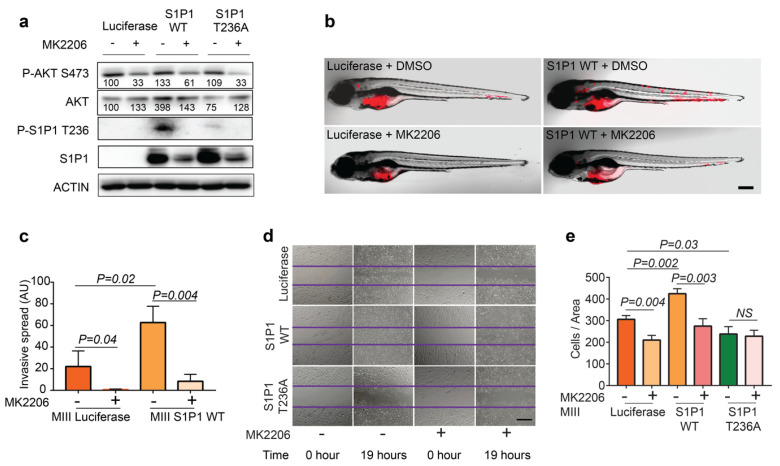
AKT-mediated phosphorylation of S1P1 T236 drives TNBC invasion. (**a**) Western blot analysis of P-AKT S473, AKT, P-S1P1 T236, and S1P1 expression in MIII cells overexpressing *Luciferase*, *S1P1 WT*, or *S1P1 T236A* in the presence or absence of 0.3 μM MK2206 treatment. P-AKT and AKT to ACTIN ratio are shown as relative values, with the vehicle-treated cells overexpressing *Luciferase* set as 100. (**b**,**c**) Wound-scratch assay (**b**) and data quantification (**c**) Reveal migration characteristics of MIII cells overexpressing *Luciferase*, *S1P1 WT*, or *S1P1 T236A* treated with DMSO or MK2206 (90 nM) at 0 and 19 h post scratching, showing that MK2206′s ability to inhibit migration of TNBC cells depends on AKT-mediated phosphorylation of S1P1 T236 (*n* = 3 per group). (**d**) Overlay of brightfield and red fluorescence images of zebrafish xenografts transplanted with RFP^+^ MIII cells overexpressing *Luciferase* or *S1P1 WT* treated with DMSO or MK2206 (0.3 μM) at 3 dpt. (**e**) Quantification of fluorescence intensity of invasive tumor cells outside the injection area (*n* = 3 per group). Scale bar = 200 µm in (**b**) and Scale bar = 5 µM in (**d**). Data are presented as Mean ± SEM.

Similar to its effect on the parental MIII cells, MK2206 treatment also reduced P-AKTS473 and P-S1P1 T236 levels in MIII cells that overexpress *Luciferase*, *S1P1 WT*, or *S1P1 T236A* (Figure 5a). Interestingly, MK2206 treatment decreased the total S1P1 in MIII cells overexpressing *S1P1 WT* or *S1P1 T236A*, compared to the vehicle-treated controls (Figure 5a). Overexpressing *S1P1 WT* and not *S1P1 T236A* led to an increased total of AKT and P-AKT S473 levels, indicating that S1P1 activated its downstream AKT signaling. We then performed a scratch-wound assay of these cells and found that MK2206 treatment significantly impeded the wound healing of MIII cells overexpressing *Luciferase*, or *S1P1 WT*, compared with the vehicle-treated cells (Figure 5b,c). Interestingly, AKT inhibition by MK2206 treatment can no longer reduce the migration of MIII cells overexpressing *S1P1 T236A* (Figure 5b,c). In zebrafish xenografts, MK2206 treatment suppressed the spread of TNBC cells overexpressing *S1P1 WT* compared to the vehicle-treated controls (Figure 5d,e and Figure S4b). We conclude that AKT mediates phosphorylation of S1P1 T236 in TNBC cells to promote their aberrant migration and distant spread, which can be dampened with the AKT inhibitor MK2206 without significantly affecting cell viability.

### 3.5. FTY720 Sensitizes TNBC Cells with Elevated P-S1P1 T236 to Apoptosis and Reduces Their Invasion

**Figure 6:**
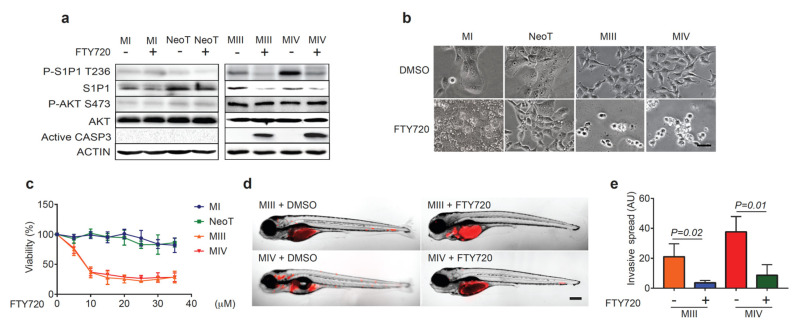
FTY720 induces apoptosis and suppresses the distant spread of human TNBC cells with high P-S1P1 T236 Levels. (**a**) Western blot analysis of P-S1P1 T236, S1P1, P-AKT S473, AKT, active CASPASE 3 (CASP3), and ACTIN levels in MI, NeoT, MIII, and MIV cells before and after FTY720 treatment (2 μM). (**b**) Brightfield images of MI, NeoT, MIII, and MIV cells at 48 h post-treatment with DMSO or FTY720 (*n* = 3 per group). The scale bar represents 1 µM. (**c**) The proportion of viable cells in response to a dose gradient of FTY720 shows a significantly reduced viability of MIII and MIV though not MI nor NeoT cells (*n* = 3 per group). (**d**) Overlay of brightfield and fluorescence images of zebrafish embryos injected with RFP^+^ MIII and MIV cells treated with DMSO or FTY720 (2 μM) at 3 dpt (*n* = 3 per group). The scale bar represents 200 µm. (**e**) Quantification of fluorescent intensity of tumor cells described in (**d**) Shows FTY720′s ability to inhibit the distant spread of TNBC cells (*n* = 3 per group). Data indicate mean ± SEM.

FTY720 is an FDA-approved drug for the first-line treatment of multiple sclerosis [32]. Phosphorylated by SPHK2 to its active form, FTY720 becomes a structural mimetic of S1P and a functional antagonist of S1P1, resulting in S1P1 downregulation through receptor internalization and degradation [15,33]. We treated the MCF10A cell series with FTY720 and performed western blot analysis to examine the total and phosphorylated S1P1 and AKT levels. We found that FTY720 treatment, though not affecting AKT S473 phosphorylation or total AKT levels, robustly reduced levels of both total S1P1 and P-S1P1 T236 in MIII and MIV though not in MI or NeoT cells (Figure 6a). Strikingly, FTY720 induced apoptosis only in MIII and MIV cells expressing high levels of P-S1P1 T236 while minimally affecting MI and NeoT cells that had low P-S1P1 T236 levels, as demonstrated by active caspase three expression and cell morphology changes (Figure 6a,b). Viability assays revealed that MIII and MIV cells, though not MI and NeoT cells, were sensitive to FTY720 treatment and had drastically reduced growth (Figure 6c).

Next, we transplanted MIII and MIV cells into zebrafish embryos, treated them with FTY720, and monitored tumor cell dissemination over time. Although fish were transplanted with similar tumor burdens (Figure S5a), FTY720 treatment significantly reduced the distant spread of MIII and MIV cells, compared to the respective vehicle-treated controls (Figure 6d,e). Immunofluorescent staining identified tumor cell apoptosis as the basis of this FTY720-induced inhibitory effect. Consistent with the reduced invasiveness of these MIII cells in vivo, FTY720 treatment significantly reduced the migration of MIIII cells in wound healing assay (Figure S5b,c). Together, our data indicate that TNBC cells with enhanced AKT-mediated phosphorylation of S1P1 T236 are particularly sensitive to FTY720, leading to increased apoptosis and reduced invasion.

## 4. Discussion

Although most patients with TNBC initially respond well to cytotoxic therapy, they rapidly develop resistance to treatment and often present with metastatic disease. When this occurs, there are limited treatment options available for these patients, underscoring the urgent need to understand the mechanisms that underlie TNBC aggressiveness in order to develop targeted therapy. Our reanalysis of a published microarray dataset showed that compared to other subtypes of breast cancer, TNBC patient samples exhibited elevated mRNA expression levels of *SPHK1* yet the decreased expression of *SPHK2*. This result challenges the concept that high *SPHK* expression results in hyperactive S1P signaling [10]. Among the five receptors for S1P, high S1P1 expression has been associated with reduced disease-specific survival and increased resistance to tamoxifen in patients with ER-positive breast cancer [21]. In the context of TNBC, others show that S1P1 contributes to the survival of tumor cells [22,23]. Our findings reveal that S1P1 is not upregulated in transcript or protein levels in human TNBC cells in comparison with the luminal subtype. Instead, more S1P1 is phosphorylated at the T236 residue in human TNBC cells. Overexpression of phosphorylation-defective *S1P1 T236A* mutant suppressed migration of TNBC cells, as demonstrated by the wound scratch assays, and distant dissemination as monitored in zebrafish xenografts. Together, our results indicated that P-S1P1 T236 contributes to TNBC aggressiveness by enhancing migration and the distant spread of tumor cells. Thus, increased phosphorylation at the T236 site of S1P1 may serve as a biomarker to predict tumor aggressiveness for TNBC patients. 

Although AKT was shown to phosphorylate S1P1 T236 and promote migration in endothelial cells [14], its regulation on S1P1 in cancer cells had not been previously investigated. Interestingly, not only did we find elevated P-S1P1 T236 in human TNBC cells but also its positive correlation with disease aggressiveness and P-AKT S473 levels. Thus, the tumor-promoting effect of S1P1 signaling in TNBC is likely to act through its downstream effector AKT, which in turn functions as an upstream regulator of S1P1 by phosphorylating its T236 residue. Indeed, we found that treatment of TNBC cells with a pan-AKT inhibitor MK2206 effectively decreased phosphorylation of S1P1 T236, inhibited tumor cell migration in vitro, and suppressed tumor cell dissemination in zebrafish xenografts. While AKT has over one hundred downstream targets [34], our studies identified S1P1 as the crucial downstream effector of AKT in TNBC, uncovering the mechanism by which hyperactivation of the PI3K/AKT pathway promotes TNBC invasion. 

Previous studies have shown that MK2206 induces apoptosis in a dose-dependent manner in certain breast cancer cell lines [31]. Additionally, MK2206 is known to inhibit the PI3K pathway, resulting in decreased expression of PD-L1: a ligand of programmed cell death protein one found in a subset of TNBC tumors [35]. However, we and others found that MK2206 minimally impacted the viability of the four human MCF10A-derived cell lines [31]. Instead, we found that a low dose of MK2206 can inhibit TNBC cell migration in vitro and distant spread in vivo as determined by using zebrafish xenografts. Others had reported that AKT3 amplification and its elevated protein expression were associated with poor recurrence-free survival in TNBC patients [36]. Hence, it is likely that AKT3 mediates phosphorylation of S1P1 T236 in TNBC cells to promote disease aggressiveness.

FTY720, the FDA-approved drug for multiple sclerosis, can effectively suppress S1P1 signaling through the downregulation of total S1P1 levels [37]. Although FTY720 was found to inhibit basal-like tumor cell growth when combined with EGFR inhibitors [11], it is unclear which subsets of TNBC patients would benefit from this treatment. Our results show that TNBC cells with elevated levels of P-T236 S1P1 are more sensitive to FTY720-induced cytotoxic effects. In addition, the treatment of human TNBC cells with FTY720 downregulated P-S1P1 T236 and inhibited tumor cell migration in vitro and disease dissemination in vivo. Indeed, FTY720 has been shown to inhibit the S1P-induced migration of classical Hodgkin lymphoma cells by modulating S1P1 expression and to reduce ovarian cancer cell migration by inhibiting SPHK1 [38,39]. Hence, FTY720 may serve as an effective treatment option for the patient population resistant to standard treatment protocols, especially those with AKT activation. Further clinical testing should be conducted to determine the benefits of including FTY720, alone or with standard therapy, for treating TNBC patients with high P-S1P1 T236 expression in their tumors, especially for those who have developed resistance to standard therapy. 

## Data Availability

The raw data will be available upon request.

## Supplementary Materials

The following supporting information can be downloaded at: https://www.mdpi.com/article/10.3390/cells12070980/s1, Figure S1: The Invasiveness of Human TNBC Cells Positively Correlates with the Expression of P-S1P1 T236; Figure S2: S1P1 T236 Phosphorylation does not Contribute to TNBC Cell Proliferation; Figure S3: MK2206 does not Impact the Viability of MCF10A Cell Series; Figure S4: MK2206 does not Impact the Viability of MCF10A Cell Series; Figure S5: FTY720 Reduces Mi-gration of TNBC Cells with Elevated S1P1 T236 Phosphorylatio; Video S1: Human MIII Cells In-travasate into Zebrafish Vasculature. RFP+ TNBC cells circulate through GFP+ vasculature of zebrafish xenografts.
