# Supplementary material for: S1P1 Threonine 236 Phosphorylation Mediates the Invasiveness of Triple-Negative Breast Cancer and Sensitivity to FTY720
Source: Cells. 2023 Mar 23;12(7):980. doi: 10.3390/cells12070980 (PMC10093541; doi:10.3390/cells12070980)
Supplement: Supplementary file 1 [file cells-12-00980-s001.zip › cells-2222327-supplementary .pdf]

Supplementary Information

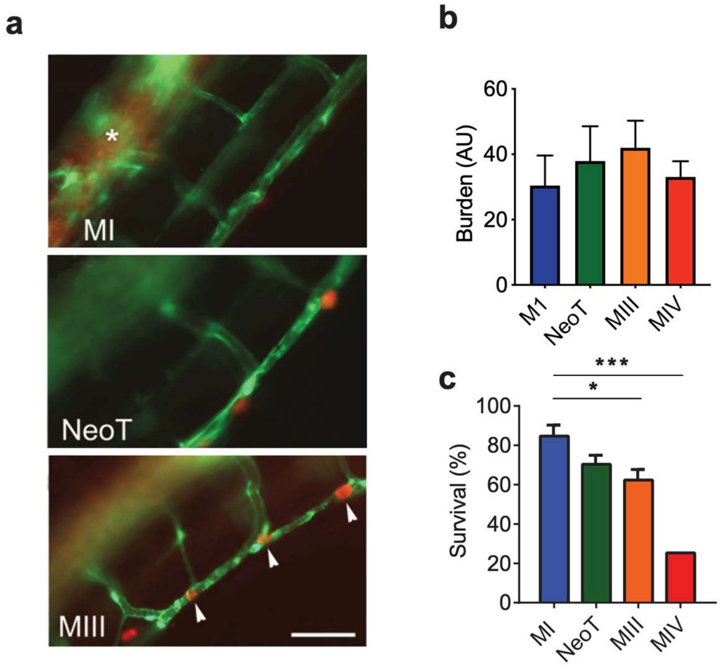

Supplementary Figure S1. The Invasiveness of Human TNBC Cells Positively Correlates with the Expression of P-S1P1 T236. (**a**) Fluorescence microscopy demonstrating intravasation of RFP^+ MIII cells, but not MI or NeoT, into the GFP^+ vasculature of zebrafish embryos at 3 dpt, as indicated by the white arrowheads. The asterisk * indicates the location of tumor cell injection. Merged images show that MIII cells (red) penetrate the zebrafish vasculature (green), while transplanted MI (red; asterisk) and NeoT cells (red cells next to the green vasculature) do not. (**b**) Quantification of fluorescence intensity reveals that the burden of transplanted MI, NeoT, MIII, and MIV cells is relatively consistent among all groups of zebrafish embryos (n=3 per group). (**c**) Proportion of viable zebrafish embryos transplanted with MI, NeoT, MIII, and MIV cells at 3 dpt (n=3 per group). Data is presented as Mean ± SEM. *$P<0.05$, *** $P<0.001$ (two-tailed Student's *t*-test).

Supplementary Video S1. Human MIII Cells Intravasate into Zebrafish Vasculature. RFP^+ TNBC cells circulate through GFP^+ vasculature of zebrafish xenografts.

https://drive.google.com/open?id=1E7rXnHA_HROsU6raNtUomndJH30QMtJ&authuser=huifeng%40bu.edu&usp=drive_fs

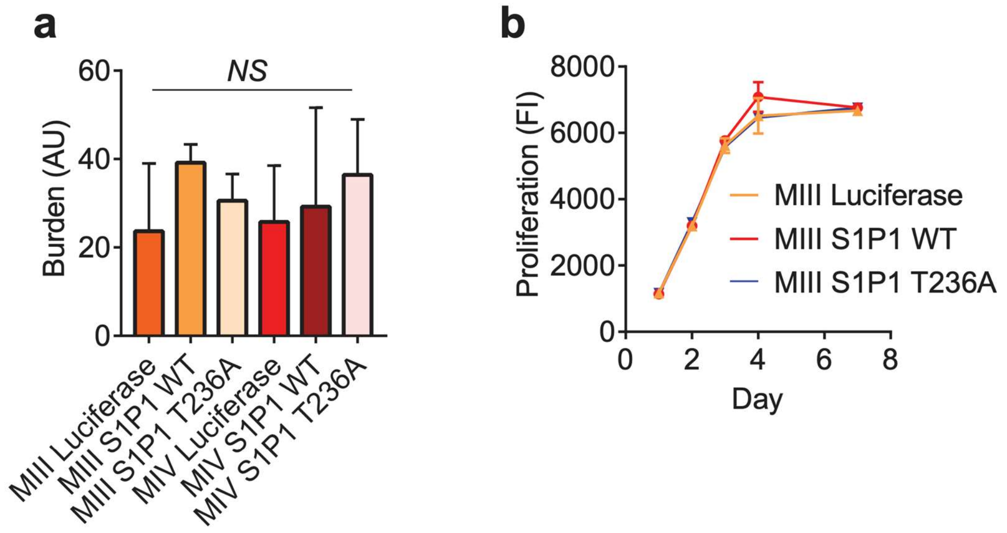

Supplementary Figure S2. S1P1 T236 Phosphorylation does not Contribute to TNBC Cell Proliferation. (**a**) Quantification of fluorescence intensity shows no significant difference in the initial tumor burden of zebrafish embryos transplanted with MIII and MIV cells overexpressing *Luciferase*, *S1P1 WT*, or *S1P1 T236A*. (**b**) Proliferative rates of MIII cells overexpressing *Luciferase*, *S1P1 WT*, or *S1P1 T236A*, showing no significant differences (n=3 per group). Data is presented as Mean ± SEM.

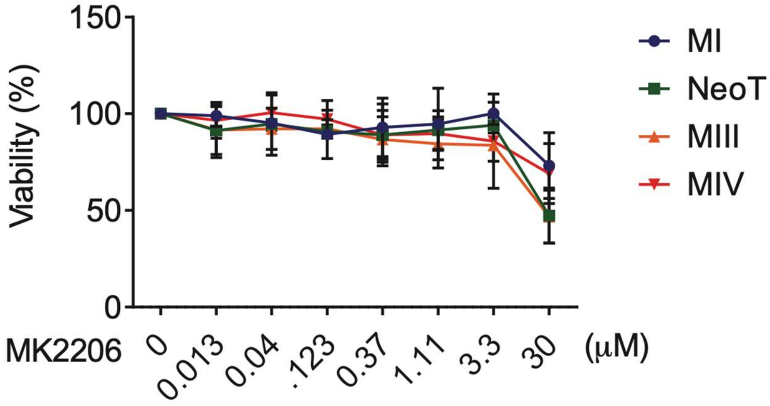

Supplementary

Figure S3. MK2206 does not Impact the Viability of MCF10A Cell Series.

Proportion of viable MI, NeoT, MIII, and MIV cells in response to a dose gradient of MK2206 (up to 30 μM) over 48 hrs, showing no significant differences among the cell lines (n=3 per group).

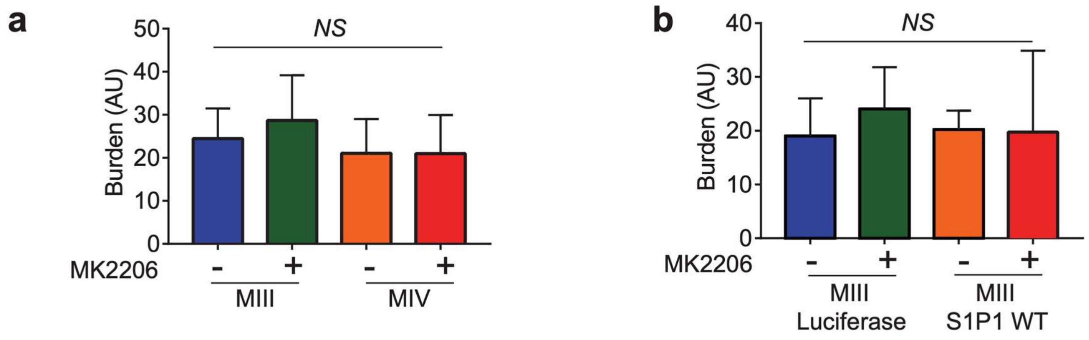

Supplementary Figure S4. Similar Tumor Burden in Different Fish Groups Before Treatment. (**a-b**) Quantification of fluorescence intensity shows no significant difference in the initial tumor burden of zebrafish embryos transplanted with MIII and MIV cells (**a**) or TNBC cells overexpressing Luciferase or S1P1 WT (**b**) treated with vehicle or MK2206 ($n \geq 3$ per group).

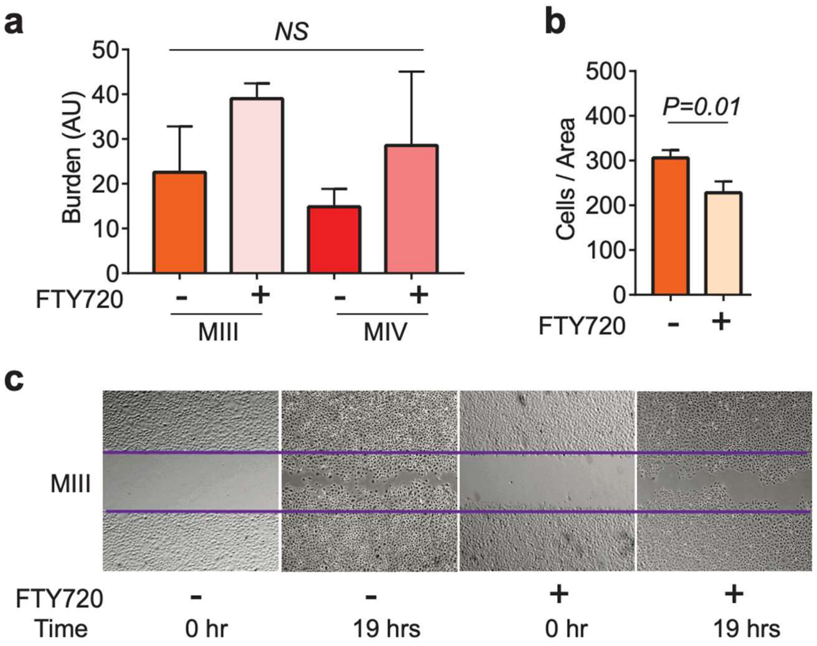

Supplementary Figure S5. FTY720 Reduces Migration of TNBC Cells with Elevated S1P1 T236 Phosphorylation. (**a**) Quantification of fluorescence intensity shows no significant difference in the initial tumor burden of zebrafish embryos transplanted with MIII and MIV cells in different treatment groups (n≥3 per group). (**b-c**) Wound healing quantification (**b**) and images (**c**) showing the migration characteristics of MIII cells treated with DMSO or FTY720 (2 μM) at time points 0 and 19 hrs. The quantification data is for MIII cells at the time point 19 hrs. Scale bar represents 500 μm. Data indicates mean ± SEM.
